# Chitosan-Functionalized-Graphene Oxide (GO@CS) Beads as an Effective Adsorbent to Remove Cationic Dye from Wastewater

**DOI:** 10.3390/polym14194236

**Published:** 2022-10-09

**Authors:** AbdElAziz A. Nayl, Ahmed I. Abd-Elhamid, Wael A. A. Arafa, Ismail M. Ahmed, Ahmed A. El-Shanshory, Mohamed A. Abu-Saied, Hesham M. A. Soliman, Mohamed A. Abdelgawad, Hazim M. Ali, Stefan Bräse

**Affiliations:** 1 Department of Chemistry, College of Science, Jouf University, Sakaka 72341, Al Jouf, Saudi Arabia; 2 Advanced Technology and New Materials Research Institute, City of Scientific Research and Technological Applications (SRTA-City), New Borg Al-Arab 21934, Alexandria, Egypt; 3 Department of Pharmaceutical Chemistry, College of Pharmacy, Jouf University, Sakaka 72341, Al Jouf, Saudi Arabia; 4 Institute of Organic Chemistry (IOC), Karlsruhe Institute of Technology (KIT), Fritz-Haber-Weg 6, 76133 Karlsruhe, Germany; 5 Institute of Biological and Chemical Systems—Functional Molecular Systems (IBCS-FMS), Director Hermann-von-Helmholtz-Platz 1, 76344 Eggenstein-Leopoldshafen, Germany

**Keywords:** graphene oxide@chitosan composite beads, removal, eco-friendly, cationic dye, wastewater

## Abstract

In this study, the preparation of graphene oxide@chitosan (GO@CS) composite beads was investigated via continuous dropping techniques to remove methylene blue (MB)-dye from an aqueous media. The prepared beads were characterized using various techniques before and after the adsorption of MB. The experimental results showed that the adsorption processes fit the kinetic pseudo-second-order and Langmuir isotherm models. Moreover, the GO@CS beads achieve maximum adsorption capacities of 23.26 mg g^−1^, which was comparable with other adsorbents in the literature. An important advantage of our adsorbent is that the GO@CS can remove 82.1% of the real sample color within 135 min.

## 1. Introduction

Several industries such as textile, pigment, paper, food, leather, etc., require dyes in their processes. These industries release a huge amount of wastewater polluted with dyes. This pollutant contains various toxic chemicals that are hazardous to the environment, animals, and humans. Moreover, these contaminants can gather in soil and cause long-term danger. Hence, proper management of these hazardous materials is required [1]. Therefore, the removal of such contaminants from wastewater is highly necessary before discharging the treated water into the environment [2]. Several techniques such as chemical oxidation, flocculation, ozonation, adsorption, reverse osmosis, etc., have been investigated to eliminate these contaminates from wastewater. However, most of these treatment processes have many disadvantages and limitations, such as cost and the generation of undesirable wastes [3]. Therefore, the adsorption technique appears to be the most promising due to its easy operation, efficiency, low-sludge production, cost-effectiveness, and simplicity of setup [3,4]. During this decade, various adsorbent materials were investigated as effective adsorbents to remove Methylene blue (MB)-dye from wastewater [5,6,7,8,9]. However, many types of these adsorbent materials have some restrictions to use in wastewater purification [7], and therefore several efforts were made to develop excellent adsorption materials with economic and eco-friendly properties derived from natural resources [7,10]. Chitosan (CS), a natural biopolymer, is an example of promising adsorbent material derived from natural resources; therefore, it has gained great attention due to its properties. The chemical structure of chitosan (CS) contains several active groups, such as amino- (-NH_2_) and hydroxyl- (-OH) groups, which serve as coordination sites for various pollutants; therefore, it is considered as an efficient adsorbent material for wastewater treatment [10]. Recently, many works have been investigated to modify the chitosan to enhance its efficiency in removing dyes and other pollutants from wastewater [2,3,7,10,11,12,13,14,15,16,17,18,19,20,21]. Modification of CS with carboxymethyl to synthesize a novel cross-linked O-CM-chitosan hydrogel adsorbent materials to remove the MB dye [2]. A novel adsorbent material, such as activated carbon@CS microbead and sponge material of karaya gum and CS, was prepared to adsorb the MB dye from wastewaters [3,7]. Also, novel polyacrylamide (PAAM)/CS) adsorbent materials were investigated as low-cost and eco-friendly bio adsorbent decolorization materials for MB dye [11]. At the current time, graphene oxide (GO) is used as adsorbent material for different pollutants from wastewater [14], with considerable amounts of oxygen-containing functional groups [14]. To enhance the adsorption capacity of CS to bind cationic dyes, which negatively charged materials could adsorb, the introduction of GO onto CS to incorporate additional carboxylic groups as active anionic sites improves its adsorption capacities to dyes from aqueous media [3,7]. As reported in the literature, GO was cross-linked with CS to prepare a composite material to adsorb MB-dye [20]. GO-CS composite aerogel aerogels was fabricated using postponed crosslinking to adsorb MB-dye [19]. Also, CS-GO composite hydrogels was investigated as an efficient adsorbent to remove MB-dye [12]. In addition, GO-CS composite was synthesized by artificial neural network-particle swarm optimization to remove MB-dye [1]. GO-lignosulfonate aerogel cross-linked with CS was investigated to enhance adsorption of MB-dye from water [21]. On the other hand, the bead forms of adsorbent materials suggested better adsorption performance compared to the other form of the composites [13].

Therefore, our goal was to prepare GO@CS beads using a simple continuous dropping technique using a peristatic pump to remove methylene blue (MB)-dye from an aqueous media. Subsequently, the obtained beads were characterized with SEM, FTIR, and TGA before and after the adsorption process. Finally, the GO@CS was applied to the treatment of the real sample.

## 2. Materials and Methods

### 2.1. Materials and Instrumentation

All chemicals used were of analytical grade and used without any purification. H_2_SO_4_ (95–97%, Riedel deHaen), H_2_O_2_ (36%, Pharaohs Trading and Import, Cairo, Egypt), HCl (30%, El Salam for Chemical Industries, Midland, MC, USA), KMnO_4_ (99%, Long live), and graphite (200 mesh, 99.99%, Alpha Aesar, Ward Hill, MA, USA), Methylene blue (Sigma-Aldrich, St. Louis, MI, USA)

Analytical balance (CP 2245, Sartorius, Göttingen, Germany), Hot plate stirrer (IKA, C-MAG HS7, IKA^®^-Werke GmbH & Co. KG, Breisgau, Germany), pH meter (3510, Genway), Hot plate stirrer (SB 162, Stuart, UK.), Centrifuge, (Mikro 220R, Hettich, Salford, UK).

### 2.2. Characterization

The synthesized GO@CS composite beads were characterized by Scanning Electron Microscope (SEM) (JEOL GSM-6610LV. Tokyo, Japan) Thermo-Gravimetric Analysis (TGA, Shimadzu Thermal Gravimetric Analysis (TGA)—50, Tokyo, Japan). Fourier Transmission Infra-Red Spectroscopy (FT-IR) (8400s, Shimadzu, Kyoto, Japan) covered the range 400–4000 cm^−1^ and Raman Spectroscopy (Bruker, Senterra II, Bremen, Germany).

### 2.3. Preparation of GO@CS Composite

**Figure 1:**
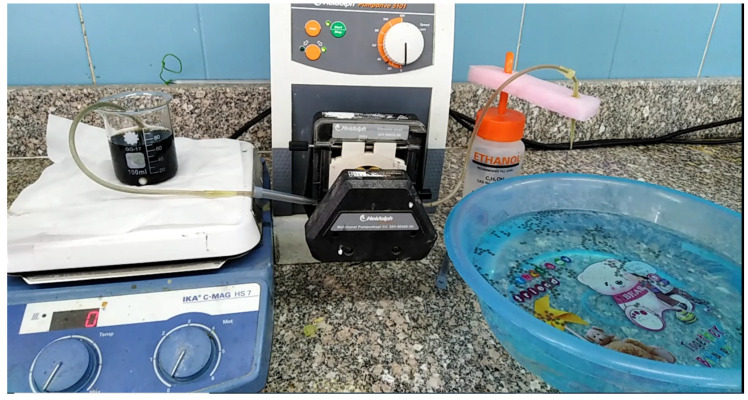
Photographic images of the continuous system used to prepare GO@CS beads.

**Figure 2:**
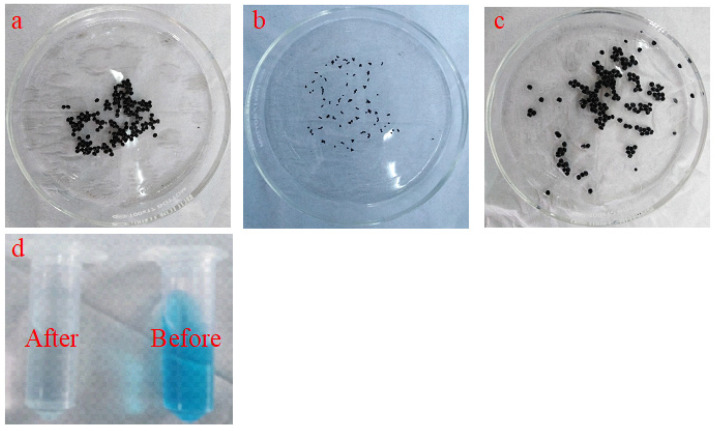
Photographic images of (**a**) as prepared GO@CS beads, (**b**) GO@CS beads after air drying, (**c**) GO@CS beads after use in the treatment process, and (**d**) the dye solution before and after the decontamination process.

Graphene oxide (GO) was prepared according to our previous work [22]. To prepare GO@CS beads, 300 mg of previously prepared GO was added to an aqueous solution of (2% CS/2% acetic acid) and stirred for 24 h to give a homogenous suspension. A peristaltic pump was employed to continuously drop homogenous suspension in 3% NaOH aqueous solution to form GO@CS beads structure. Figure 1 shows the photographic image of the continuous dropping system. The formed GO@CS beads were left for 24 h in the NaOH solution for complete cross-linking. After that, it was separated by a strainer, washed several times with distilled water, and air dried for further use. Figure 2 represents the photographic image for (a) prepared GO@CS beads, (b) GO@CS beads after air drying, (c) GO@CS beads after the treatment process, and (d) the dye solution before and after the decontamination process.

### 2.4. Batch Adsorption

In this experiment the initial MB-dye concentration of 10–50 mg/L were employed. The pH of the MB-dye solution varied from 1.7 to 12. Various doses of adsorbents (0.025–0.125 mg). A temperature in the range of 35–80 °C was applied. The Effect of the NaCl dose was in range (0.0–1.0 g). Finally, 0.5 mL of the dye solution was isolated and centrifuged. After phase separation 0.1 mL of the centrifuged solution was diluted to 10.0 mL with distilled water and MB-dye concentrations were measured at 662 nm.

The dye removal percent (%R) is defined as:

%R = (C_o_ − C_t_)/C_o_ × 100 (1)

where, C_o_ and C_t_ are the initial concentrations and the concentrations of dye at time t, respectively.

### 2.5. Kinetic Models

Adsorption Kinetic models investigated in this work were represented in the Supplementary Material file (Tables S1 and S2).

## 3. Results and Discussion

### 3.1. Characterization

#### 3.1.1. SEM

**Figure 3:**
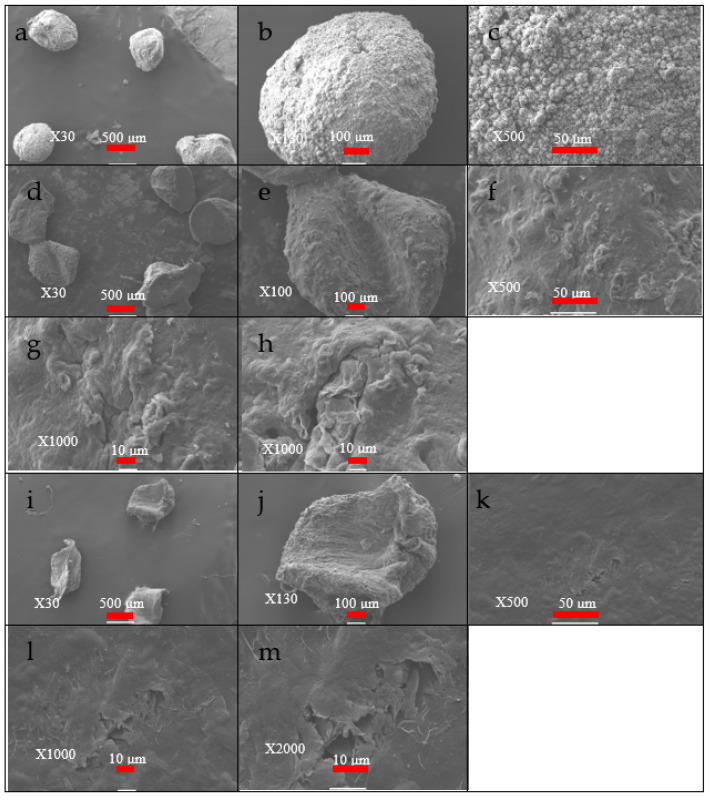
SEM images of, (**a**–**c**) the GO@CS beads, (**d**–**h**) the GO@CS beads after one cycle use, and (**i**–**m**) the GO@CS beads after five cycle use.

The SEM images in Figure 3a–c show that the dripping technique produces semi-spherical gel beads after falling the GO@CS solution in NaOH solution, Figure 3b. The surface of the beads was shown to be a non-homogenous structure. This rough surface increases the surface area, enhancing adsorption efficiency, as in Figure 3c. After the first reusing process, the roughness degree of the GO@CS surface beads reduced, which may be due to treating with HCl for desorbing the adsorbed species, as represented in Figure 3h. The beads’ surface becomes highly smooth after five reuse runs, as shown in Figure 3i–m.

#### 3.1.2. FTIR

**Figure 4:**
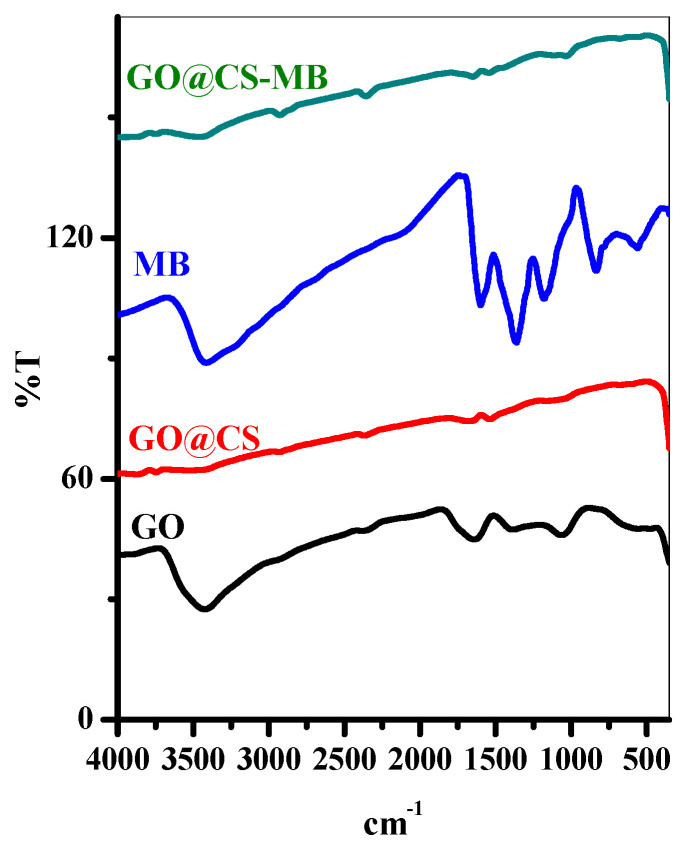
FTIR patterns of GO, GO@CS beads, MB and GO@CS beads-MB complex.

The FTIR is considered an efficient tool for assessing different functional groups in the analyzed materials. Figure 4 shows the FTIR spectra of GO, GO@CS, MB, and GO@CS-MB complexes. The GO shows a characteristic peak at 3433 cm^−^^1^ corresponding to surface adsorbed water, 1730 cm^−^^1^ related to the carboxylic group, 1629 cm^−^^1^ referring to the bending motion of water molecule, 1394 cm^−^^1^ corresponding to the deformation of the carbonyl group and finally, peak at 1064 cm^−^^1^ attributed to C-O bond stretching [22]. Accordingly, the FTIR spectrum highly differs in mixing GO and CS to form the beads. The GO/CS shows peaks at 2931 cm^−^^1^ related to C-H stretching motion, 1669 cm^−^^1^ due to water bending motion, and 1535 cm^−^^1^ due to chitosan N-H bond stretching [23]. After adsorption of MB over the GO@CS beads, the peaks’ GO@CS-MB complex shows a red shift compared with the GO@CS spectrum, which suggests a successful adsorption MB dye process [24].

#### 3.1.3. TGA

**Figure 5:**
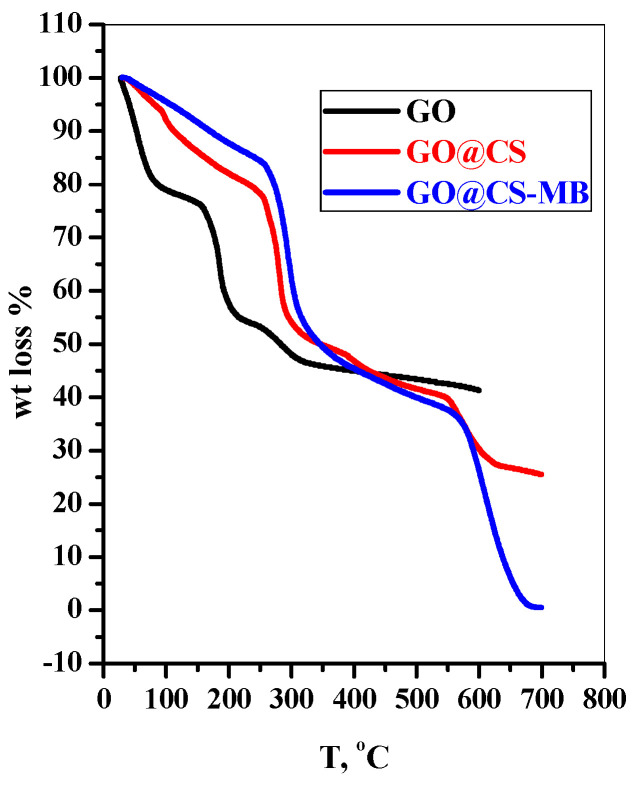
The thermogravimetric analysis (TGA) of GO, GO@CS beads, and GO@CS beads-MB complex.

The thermogravimetric analysis (TGA) is a device required to determine the thermal stability of the materials. The GO shows five decomposition stages related to the surface moisture (19.6%, 28–89 °C), interlayer water (4%, 89–158 °C), hydroxyl groups (20.5%, 158–214 °C), carboxyl groups (8%, 214–320 °C), and pyrolysis of carbon backbone (5%, 320–600 °C). With the mixing of GO and CS in beads, the amount of adsorbed moisture was highly reduced (6%, 32–92 °C), and the amount of interlayer water was enhanced due to gel formation and released at higher temperature (14%, 92–23 °C) [25]. After that, the main decomposition stage is reached at (22%, 235–288 °C) due to pyrolysis of glycosidic units of chitosan. This thermal stability achieved for the GO@CS beads may be due to the electrostatic interaction among the -NH_3_^+^ in CS and COO^−^ in graphene [25,26]. After adsorption of the MB dye species, the thermal stability of the beads collapsed and recorded two main thermal decomposition stages (35%, 235–357 °C) and (36%, 561–700 °C), as represented in Figure 5. This phenomenon may be due to consuming different function groups in the adsorption process, weakening the electrostatic attraction between the bead’s precursors.

### 3.2. Adsorption Study

#### 3.2.1. Effect of Contact Time

**Figure 6:**
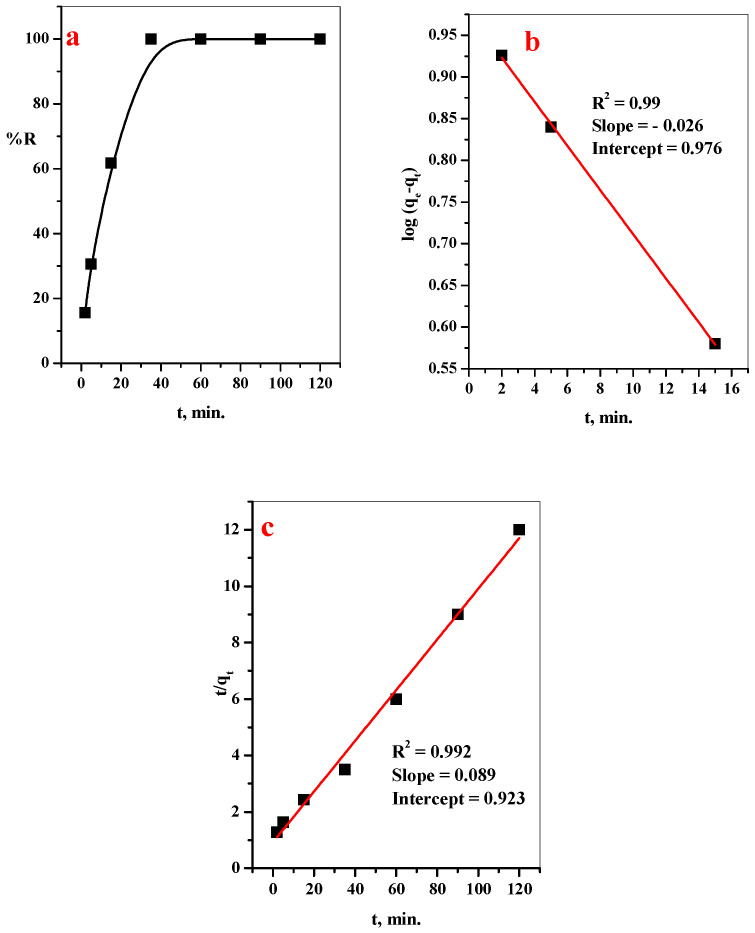
Effect of (**a**) contact time on removal percentage, (**b**) Pseudo-first-order, (**c**) Pseudo-second-order plots of the MB-dye over GO@CS composite beads. ([MB] = 10 ppm, [GO@CS] = 50 mg/50 mL, pH = 7, T = 25 °C).

A graph percent vs. stirring time at a stable of the other condition is plotted in Figure 6a. The plots show an increase in the removal percent (%R) over the contact time, and the highest adsorption efficiency is 99.9% at 35 min. After that, the removal percent becomes stable until 120 min. This behavior is attributed to the occupation of the active sites with the MB-dye molecule, and the adsorbent appearing to be saturated with the pollutant [27].

To understand the kinetic mechanism of the adsorption of MB-dye using GO@CS beads, different adsorption kinetic models (Pseudo-first- and second-order) are investigated and represented in Figure 6b,c. Different parameters and correlation coefficients related to the kinetic models are calculated from the slopes and intercepts from the linear relations in Figure 6b,c and summarized in Table 1, since R^2^ is the most utilized metric to determine the suitable fit kinetic models [1]. The relation coefficient related to the pseudo-second-order kinetic model (R^2^ = 0.992) is greater than the pseudo-first-order kinetic model, indicating that the pseudo-second-order kinetic model better fits the adsorption of MB-dye over GO@CS composite beads.

#### 3.2.2. Effect of initial MB-Dye Concentrations

**Figure 7:**
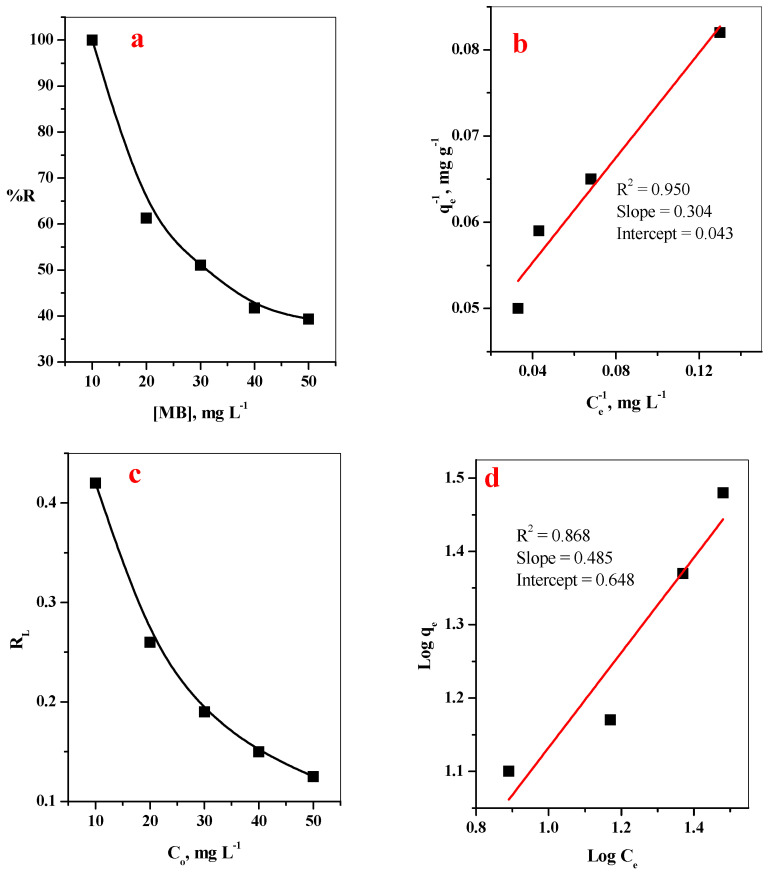
Effect of (**a**) dye concentration on removal percent, (**b**) Langmuir isotherm plot, (**c**) R_L_, (**d**) Freundlich isotherm plot of MB dye. (t = 35 min, [GO@CS] = 50 mg/10 mL, pH = 7, T = 25 °C).

The initial MB-dye concentrations induced were studied in range 10–50 mg/L, as investigated in Figure 7a. The results shows that the %R of MB over GO@CS composite beads was reduced from 99.99–39.3% with a further increase in the MB-dye concentrations from 10 to 50 mg/L. This observation is attributed to the supposition that, at low MB-dye concentration, the number of MB-dye species will be equivalent to the number of the binding sites on the surface of GO@CS composite beads [28]. With increased dye concentration, the number of dye species in the aqueous solution increase compared with the number of the binding site, leading to reduced adsorption performance [29]. To explain how the dye species interact with the GO@CS beads binding sites, various adsorption isotherm models are validated (Table 2). The linear relations of the studied models are plotted in Figure 7b–d. The slopes and intercept of the fitted lines were used to calculate different isotherm parameters and listed in Table 2 as well as the relation coefficient. Based on the obtained data, the R^2^ related to Langmuir isotherm is greater than Freundlich isotherm. It is indicated that the MB-dye prefers to adsorb on an equally energetic binding site as a monolayer on the GO@CS beads surface with a maximum adsorption capacity of 23.26 mg g^−1^.

#### 3.2.3. Effect of GO@CS Composite Beads Dose, NaCl Dose, and Temperature on %R of MB-Dye

**Figure 8:**
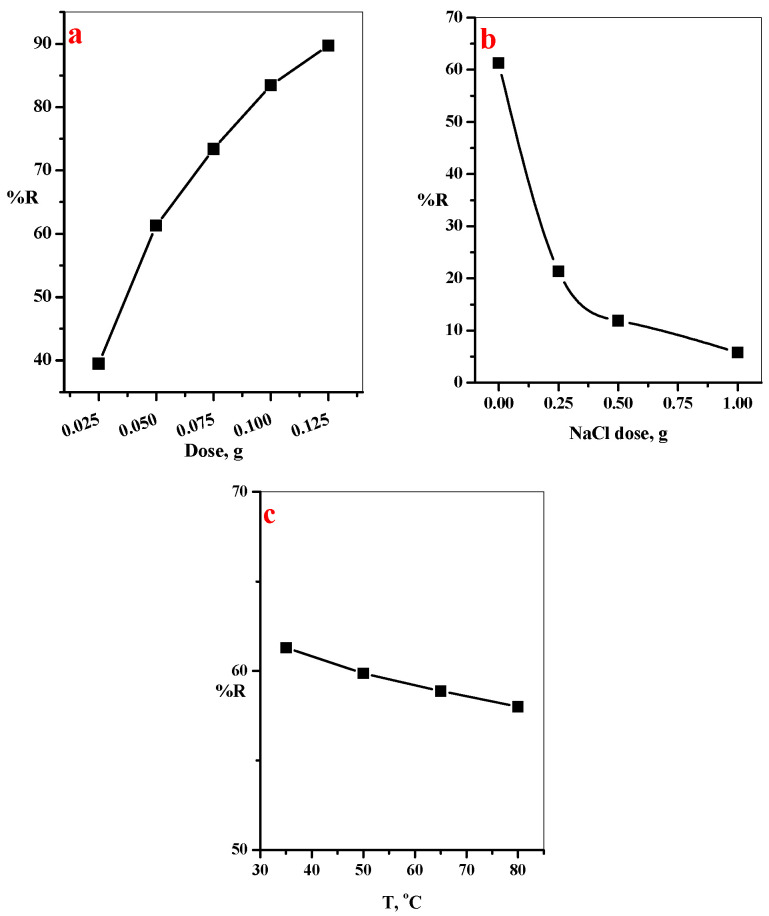
Effect of (**a**) GO@CS beads dose/50 mL dye solution (t = 35 min, [MB] = 20 mg/L, pH = 7, T = 25 °C, (**b**) NaCl dose (t = 35 min, [GO@CS] = 50 mg/50 mL, [MB] = 20 ppm, pH = 7, T = 25 °C), and (**c**) temperature (t = 35 min, [GO@CS] = 50 mg/50 mL, [MB] = 20 ppm, pH = 7) on removal percentage of MB-dye onto GO@CS composite beads.

Figure 8a represents the influence of GO@CS composite beads dose on the removal efficiency of MB-dye. It is noted that the adsorption performance of MB-dye was enhanced with further increase in the GO@CS composite beads dose. This is attributed to the further increase in the adsorbent dose, providing different binding sites for successful interaction with more MB-dye species [30]. The NaCl dose induced on %R of MB-dye was investigated in the range of 0.0–1.0 g, as shown in Figure 8b. The experimental data shows a reduction in the adsorption percentage of MB-dye from 61.29 to 5.80%, with a further increase in the NaCl dose from 0.0 to 1.0 g. This may be because the presence of Na^+^ ions in the aqueous medium will compete with the MB dye on the binding sites, leading to decreased adsorption efficiency [31].

The effect of temperature shows a very slight influence on the adsorption efficiency of MB dye, as noted in Figure 8c, that a further increase in the temperature from 25 to 80 °C leads to a decrease in the removal percentage from 61.9 to 58%. This indicated that the adsorption process was an endothermic process.

#### 3.2.4. Effect of pH on the Removal Percent of MB-Dye

**Figure 9:**
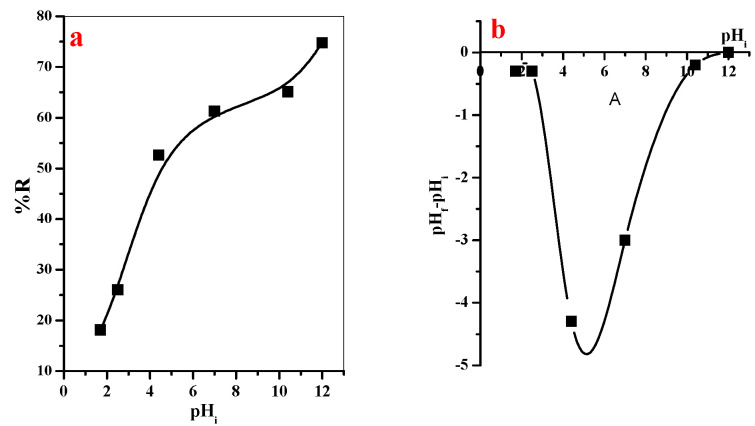
(**a**) Effect of pH on adsorption percentage of MB-dye (t = 35 min, [GO@CS] = 50 mg/50 mL, [MB] = 20 ppm, T = 25 °C), (**b**) point of zero charge of GO@CS beads.

The pH is a significant parameter to study adsorption processes. This is due to the pH of the aqueous medium highly affected on the ionization of GO@CS composite beads binding sites. Here, the influence of initial solution pH on the adsorption efficiency of MB-dye from aqueous media was investigated and represented in Figure 9a. It is interestingly observed that the increase in the pH of the solution in range 1.7–12 is accompanied with a linear increase in the adsorption efficiency from 18.1 to 74.8%. This is because, in acidic environment, the binding site will be protonated, and thus considered unavailable for adsorption of cationic MB-species [32]. With an increase in the solution pH value, the concentration of H^+^ will be reduced and the binding sites become more ionized, which provides a suitable environment for efficient removal of cationic MB-dye.

The final pH (pH_f_) of the adsorption solution was assessed for calculation of ΔpH (pH_f_—pH_i_). Thereafter, a relation was plotted between ΔpH vs. pH_i_ to determine the point of zero charge related to the GO@CS, as seen in Figure 9b. The graph showed that the adsorbent was negatively charged, even if it located in a highly acidic environment.

### 3.3. Reusability Study

**Figure 10:**
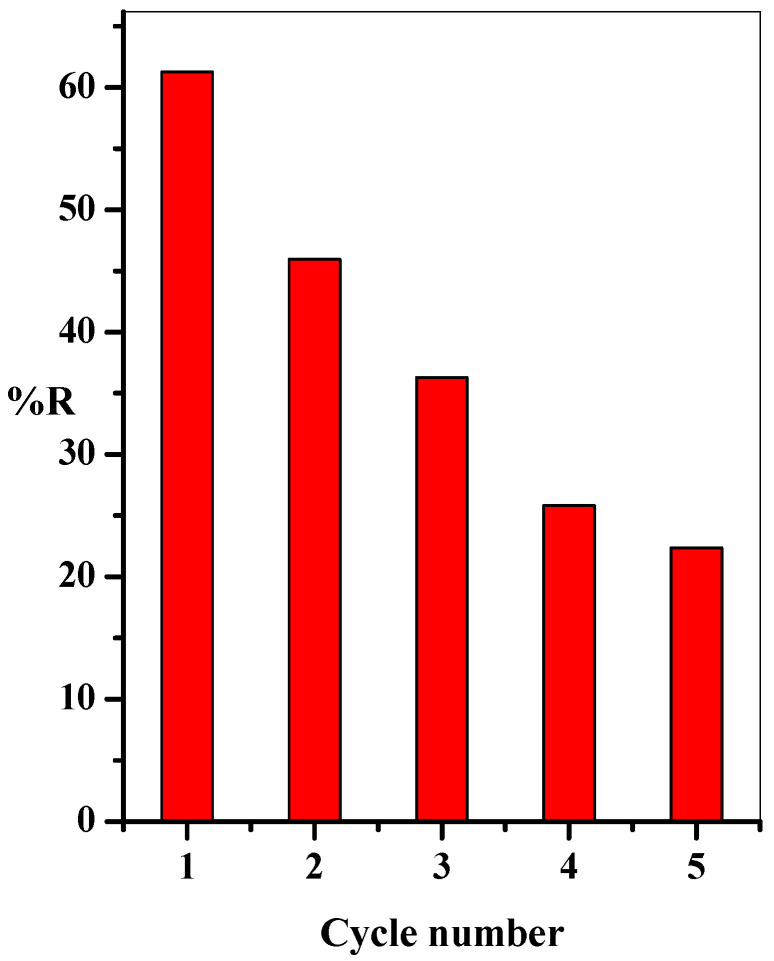
Effect of cycle number on the adsorption percentage of MB-dye (t = 35 min, [GO@CS] = 50 mg/50 mL, [MB] = 20 mg/L, pH= 7, T = 25 °C).

The reusability of the GO@CS beads was investigated for economic applications. Here, the optimum adsorption conditions extracted from the batch investigation were employed to study the adsorbent’s recyclability, as shown in Figure 10. First, the adsorbent was stirred with the aqueous dye solution for a definite time, separated by decantation, and the residual concentration of the dye was assessed. Finally, the GO@CS beads were washed with (5 mL, 1.0%) HCl solution. A decrease in the adsorption efficiency from 61.3 to 22.4% after 5 reused runs was noted. This may be blocking some binding sites by highly interacted dye species leading to occupying these binding sites and making them unavailable for further adsorption.

### 3.4. Application on the Real Dye Sample

**Figure 11:**
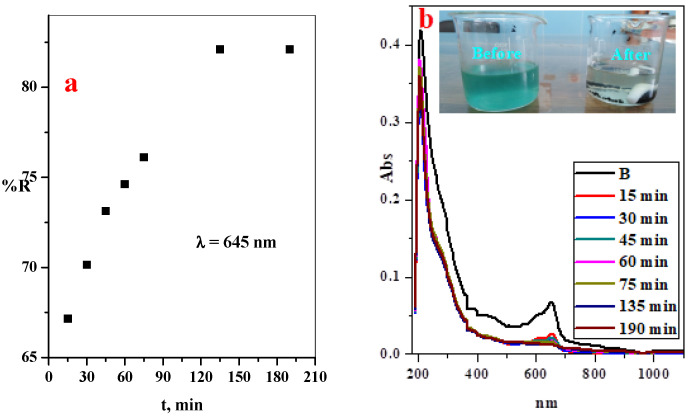
(**a**) Effect of contact time on the adsorption percentage of real sample dye using GO@CS composite beads and (**b**) the UV-Vis spectrum of the MB-dye from real sample at different contact time with GO@CS composite beads, inset photographic image of the factory dye before and after the treatment processes.

For practical application, a real sample was collected from dye factory and the dye concentration in the real sample was followed as function of the time, as illustrated in Figure 11. It was observed that the removal efficiency of the factory dye increase with the contact time and the GO@CS beads can remove 82.1% of the factory dye within 135 min.

### 3.5. Comparison Study

Table 3 represents the comparison study of the maximum adsorption capacities of MB-dye over GO@CS beads with other different adsorbents materials according to the Langmuir isotherm model published recently [1,12,13,33,34,35,36,37,38,39,40,41]. It can be observed that the GO@CS composite beads have resulted in higher MB-dye adsorption capacity compared to other adsorbents.

## 4. Conclusions

This work synthesized graphene oxide@chitosan (GO@CS) composite beads using continuous dropping techniques to adsorb methylene blue (MB) dye from wastewater. The fabricated (GO@CS) composite beads were characterized using different techniques such as SEM, TGA, FTIR, and XRD to investigate the physical and chemical properties of the prepared (GO@CS) composite beads. Under the optimum conditions, the maximum adsorption capacity was 23.26 mg g^−1^. The investigated adsorption processes obey pseudo-2nd-order and Langmuir isotherm models. A regeneration and application study on a real sample of (GO@CS) composite beads showed that it has significant adsorption efficiencies for MB-dye. Therefore, it is considered an eco-friendly adsorbent material with promising properties to use in wastewater’s decontamination processes.

## Figures and Tables

**Table 1:** Calculated parameters of the pseudo-first-order and pseudo-second-order kinetic models for adsorption of MB-dye over GO@CS composite beads.

Dye	q_e exp_ (mg/g)	Pseudo-First-Order Kinetic Parameter			Pseudo-Second-Order Kinetic Parameter		
		K_1_ (min^−1^)	q_e cal_ (mg/g)	R^2^	K_2_ (g mg^−1^ min^−1^)	q_e cal_ (mg/g)	R^2^
MB	9.99	−0.06	9.46	0.990	0.008	11.23	0.992

**Table 2:** Constants of Langmuir and Freundlich models for adsorption of MB-dye onto GO@CS composite beads.

Dye	Langmuir Isotherm Model				Freundlich Isotherm Model		
	q_e_ (mg/g)	b (L/mg)	R_L_	R^2^	*n*	K_f_ (mg/g)	R^2^
MB	23.26	0.14	0.42	0.950	2.06	4.45	0.868

**Table 3:** Langmuir based maximum adsorption capacity of several adsorbents for MB-dye adsorption.

Adsorbent	Adsorption Capacity (mg/g)	Reference
GO-CS composite	7.53	[1]
CS-GO hydrogel	14.31	[12]
CS-CNT hydrogel	21.74	[12]
Chitosan-Clay Biocomposite Beads	2.385	[13]
alginate graft-polyacrylonitrile beads	3.79	[33]
H_2_SO_4_ crosslinked magnetic chitosan nanocomposite beads	20.40	[34]
(CMC)/k-carrageenan (kC)/activated montmorillonite (AMMT) beads	12.5	[35]
Magnetic Biochar	22.88	[36]
Desmodesmus sp. Immobilized Alginate beads	20	[37]
Alginate-grafted-poly (methyl Methacrylate)	5.25	[38]
P-N-LDHs hydrogel	6.03	[39]
Silsesquioxane-based disulfide-linked polymer (DLP)	12.90	[40]
Chitosan/laterite/iron oxide	16	[41]
GO@CS beads	23.26	This work

## Data Availability

Data is contained within the article.

## Supplementary Materials

The following supporting information can be downloaded at: https://www.mdpi.com/article/10.3390/polym14194236/s1, Table S1. Kinetic model, Table S2. Linear forms of adsorption isotherm models. References [42,43] are cited in the supplementary materials.
