# Supplementary material for: Chitosan-Functionalized-Graphene Oxide (GO@CS) Beads as an Effective Adsorbent to Remove Cationic Dye from Wastewater
Source: Polymers (Basel). 2022 Oct 9;14(19):4236. doi: 10.3390/polym14194236 (PMC9572660; doi:10.3390/polym14194236)
Supplement: Supplementary file 1 [file polymers-14-04236-s001.zip › polymers-1932276-supplementary.pdf]

Experimental Section

Kinetic Models

Table S1. Kinetic model [42].

Isotherm	Linear form	Plot	Slope and intercept
1st order model	$\log(q_e - q_t) = \log q_e + \frac{K_{ads}t}{2.303}$	$Log(q_e\text{-}q_t)$ vs t	Slope = $K_{ads}/2.303$<br>Intercept = log q_e
2nd order model	$\frac{t}{q_t} = \frac{1}{K_2 q_e^2} + \frac{t}{q_e}$	t/q_t vs t	Slope = $1/q_e$<br>Intercept = $1/K_2q_e^2$

Table S2. Linear forms of adsorption isotherm models [42,43].

Isotherm	Linear Form	Plot	Slope and Intercept
Langmuir	$\frac{C_e}{q_e} = \left(\frac{1}{Q_o b}\right) + \left(\frac{1}{Q_o}\right) C_e$	C_e/q_e versus C_e	Slope= 1/ Q_o<br>Intercept =1/ Q_o b
Freundlich	$log\ q_e = log\ K_f + \frac{1}{n} log\ C_e$	log q_e versus log C_e	Slope= 1/n<br>Intercept = logK_f
Temkin	$q_e = BlnK_t + BlnC_e$	q_e versus lnC_e	Slope= B<br>Intercept = B ln K_T
